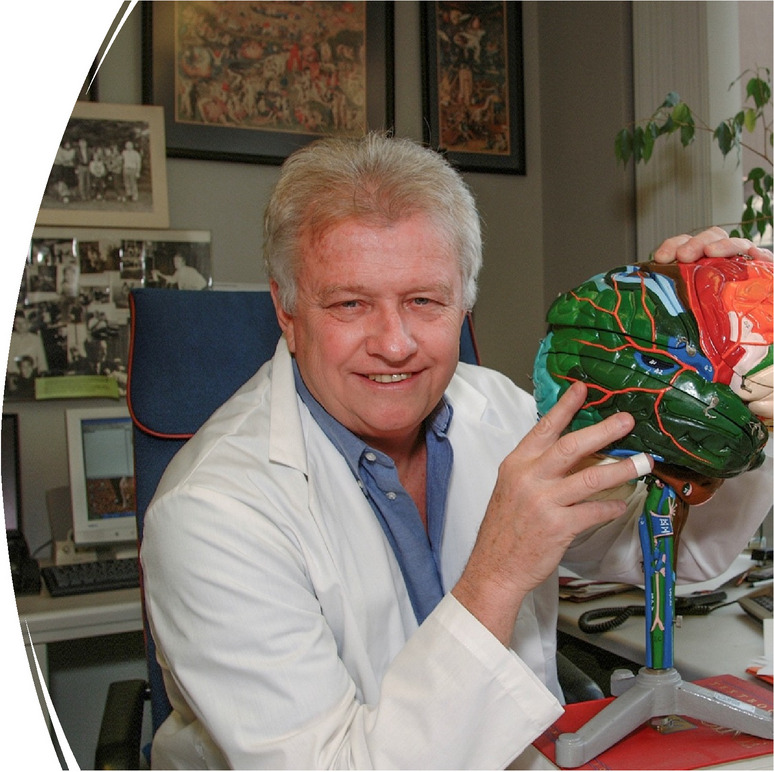# Donald Lowell Price, M.D. In memoriam

**DOI:** 10.1186/s13024-023-00670-z

**Published:** 2023-11-20

**Authors:** Sangram Sisodia, Philip C. Wong

**Affiliations:** 1 Departments of Neurobiology and Neurology, Center for Molecular Neurobiology, The University of Chicago, Chicago, USA; 2 Johns Hopkins School of Medicine, Baltimore, USA


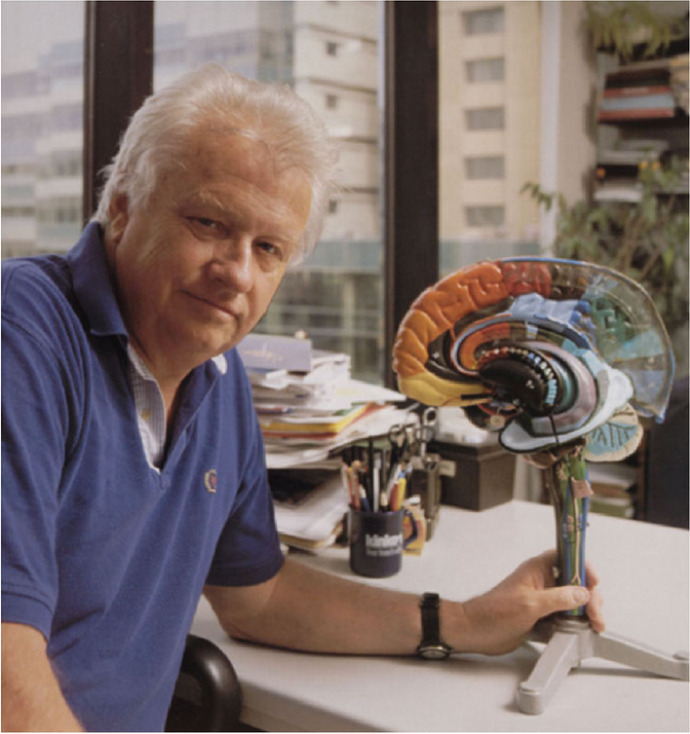


Dr. Donald L. Price, one of the most prolific and distinguished neuroscientists in the field of Alzheimer’s disease and neurodegeneration, has passed. Don was a pioneer in clinical and experimental neuropathology, a devoted husband, father and grandfather, and a mentor to a legion of scientists who followed him. Don was a quintessential renaissance man who read literature in college and thereafter, developed a keen appreciation of classical music, opera and the fine arts. After a short hiatus in minor-league baseball, he then attended Albany Medical School where he received his medical degree in 1961. From there, Don completed his residency in Neurology under the aegis of Dr. Ray Adams, and fellowship training in neuropathology with Dr. E.P. Richardson at the Massachusetts General Hospital and a research fellowship with the cell biologist and Nobel laureate, Dr. Keith Porter.
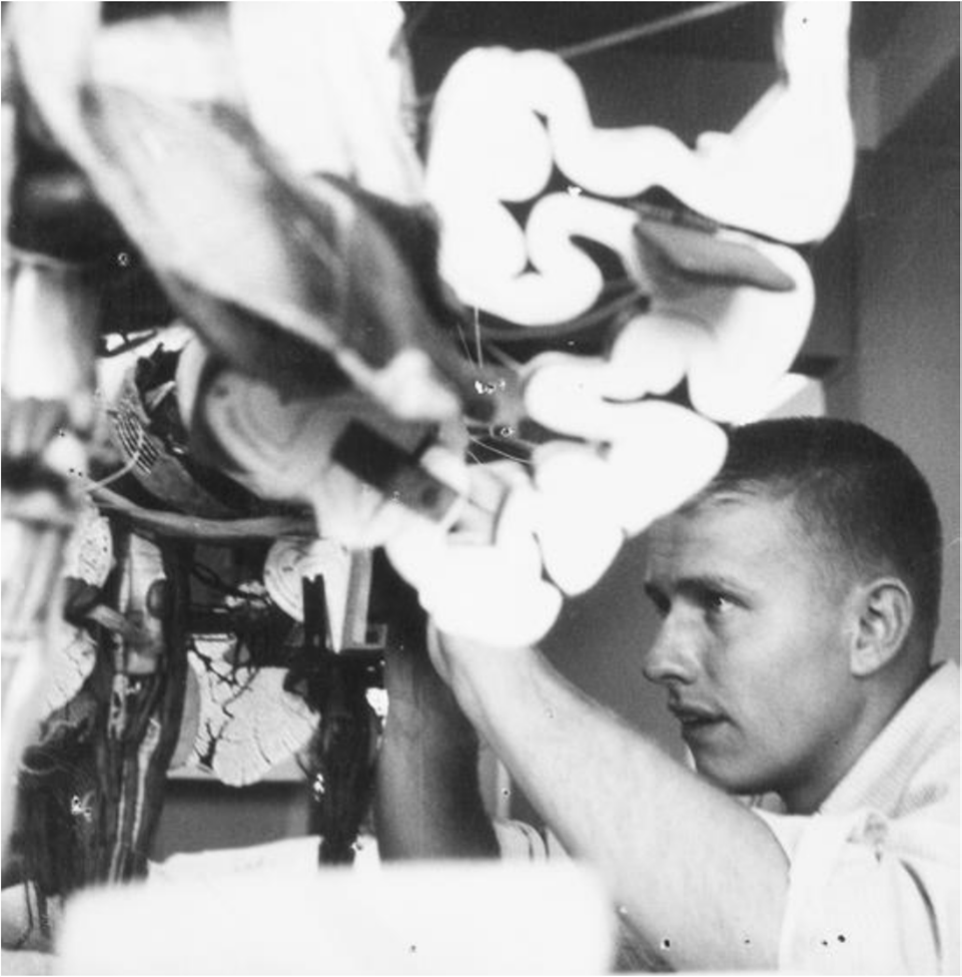


**Medical Student at Albany** 
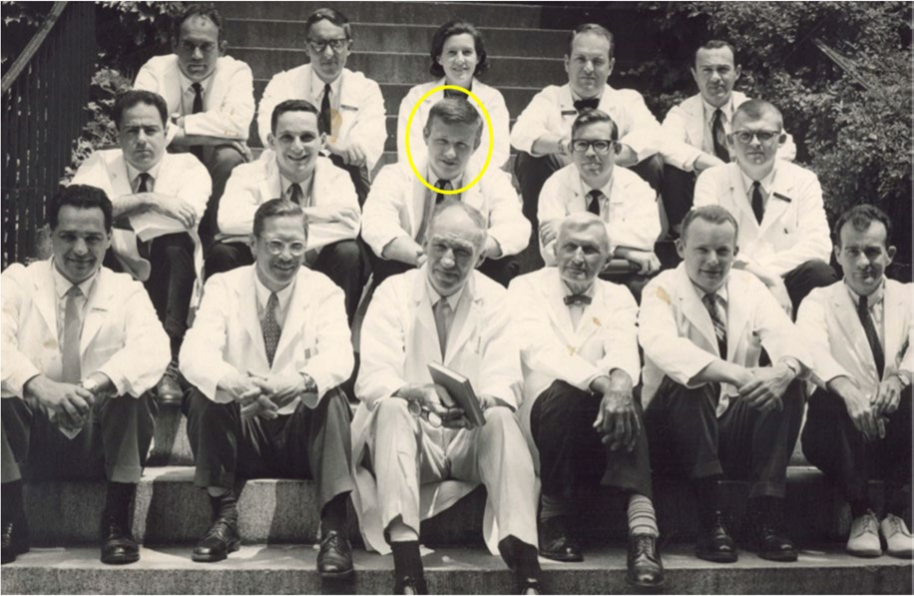


**Fellow in Neuropathology at Mass General**

Recruited to Johns Hopkins University School of Medicine in 1971, Don was the founding director of the Division of Neuropathology, which he led for four decades. As founding director of the Johns Hopkins Alzheimer’s Disease (AD) Research Center in 1984, Don and his colleagues had a significant and long-lasting impact on AD research for the next 30 years. His pioneering research efforts, driven by collaborative teams at Hopkins that led to discoveries related to cholinergic dysfunction in AD, the fields of axonal transport, comparative neuropathology and aging in nonhuman primates, as well as the development of mouse models of neurodegenerative disease, were unprecedented.

To his towering science, Don’s largest impact was in the education and training of hundreds of fellows, residents, graduate and medical students, many of whom have gone on to illustrious careers in academia and medicine. Our scientific interactions with Don focused on the development of cellular and mouse models to clarify the molecular and cellular biology of APP, presenilins and BACE1 and the underlying neurobiological mechanisms that cause AD. We both were true beneficiaries of his wisdom and kindness, and we are truly indebted.

In addition to his role as the Division head of Neuropathology at Hopkins, Don served as President of the American Association of Neuropathologists in 1989 and President of the Society for Neuroscience in 2000.
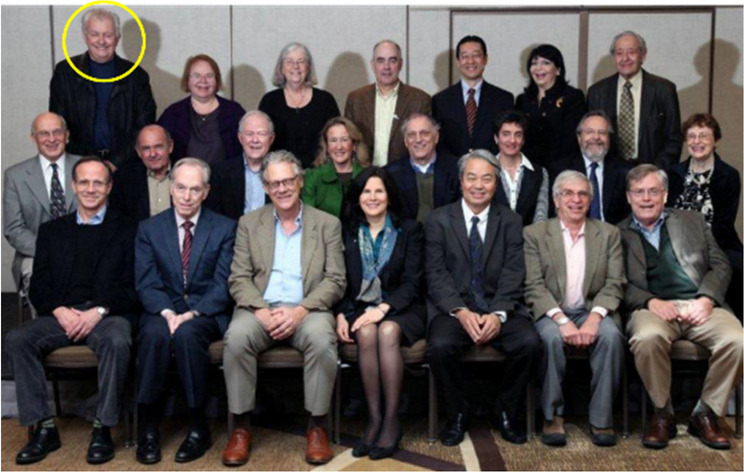


**Past Presidents of SFN** 
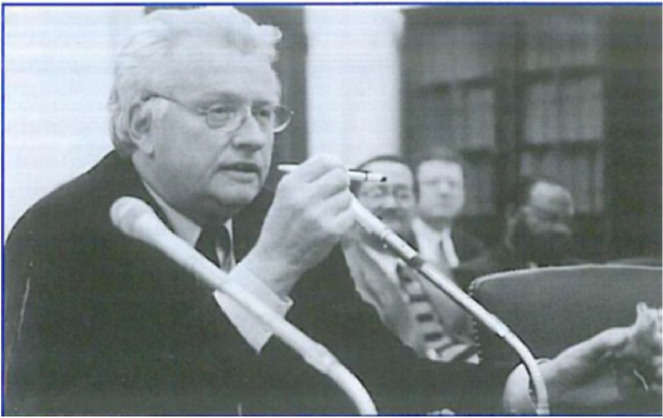


**Testifying in support of NIH budget increase as President of SFN** 
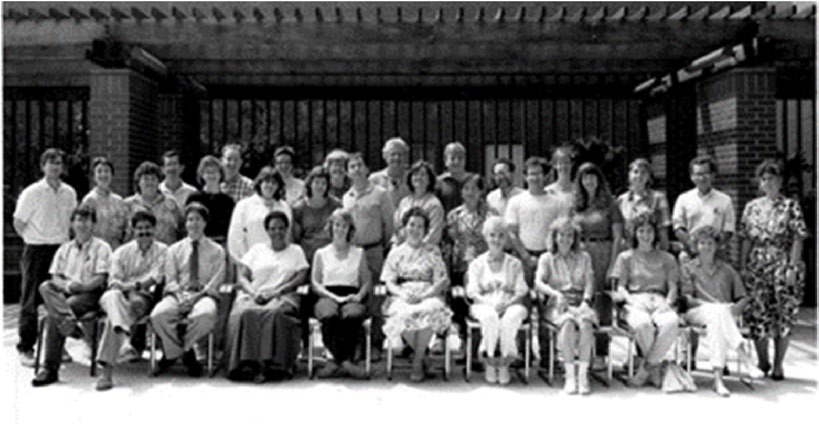


He is the author of more than 500 research publications, and during the ‘‘Decade of the Brain’’ (1990–2000), he was ranked among the top 10 neuroscientists as authors of high-impact articles by Science Watch. His work has been recognized by numerous awards, including the Bristol Myers Squibb Prize, Metropolitan Life Award for Medical Research, the Potamkin Prize for AD Research, the AAIC Lifetime Achievement Award in AD and election to the Institute of Medicine.
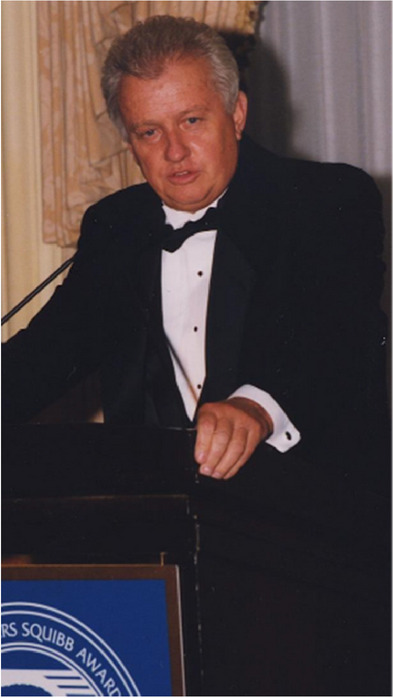


**Receiving the BMS Prize** 
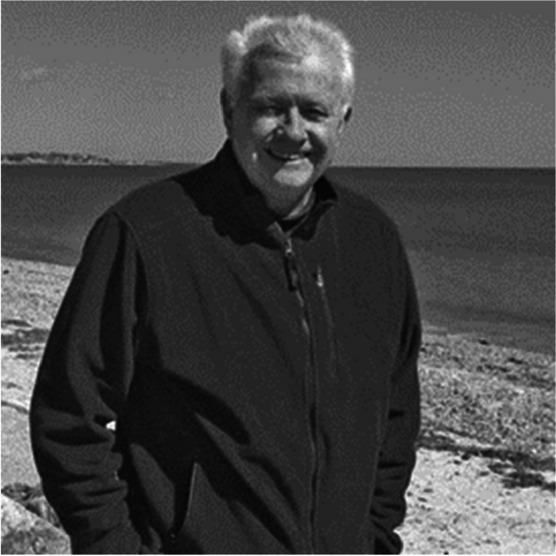


**Enjoying time at Woods Hole**

Don will be dearly missed by all who have crossed his path. His vision, wisdom and generous spirit will continue to flourish through many of the lives he so profoundly impacted.

Sam S. Sisodia, Ph.D.

Thomas Reynolds Sr. Family Professor of Neurosciences

Director, Center for Molecular Neurobiology

Departments of Neurobiology and Neurology

The University of Chicago

Phil Cy Wong, Ph.D

Professor of Pathology and Neuroscience

The Johns Hopkins University School of Medicine

Every generation in science has what we would now call an “influencer”. Don Price was the “influencer” with the most profound effect on research into Alzheimer’s Disease over the past 50 years. At a time when our view of the disease was fixated on the pathological changes, Don brought a truly neurobiological perspective. Trained in neurology, neuropathology and in cell biology, he was the first to integrate the role of neuronal communications and cellular biology with pathology in the evolution of the disease. His lab attracted a long string of outstanding young scientists. In each case, he focused as much on fostering their careers and their work as he was on his own.

I always treasured my meetings, with Don. Our shared morning runs while serving on a variety of committees were chances to discuss science, life, and the dangers of flying on airplanes. He influenced my thinking and I think that these ideas passed to my trainees who amplified what I learned from him. I will miss him.

Michael L. Shelanski, MD, PhD

Sr. Vice-Dean for Research

Henry Taub Professor of Pathology and Cell Biology

Vagelos College of Physicians and Surgeons

Columbia University

Don was a preeminent pioneer and lead thinker in the field of neurodegeneration. HIs many research contributions over many years helped launch the modern era of animal modeling and mechanistic investigations into the pathophysiology of Alzheimer's disease and other neurodegenerative diseases. Beyond that, he was a wise and just man, and a warm colleague and friend. I will always be grateful for the valuable advice he gave me at multiple stages of my career. I recall vividly the Dahlem conference he co-organized in Berlin in 1990, less than a year after the Berlin Wall had come down, which was influential in shaping my laboratory studies at Washington University. Later when I moved to work in industry on drug treatments, he continued to be someone whose judgement and counsel I sought, and which he always shared generously. It was my privilege to work beside him on the Executive Committee of the Society for Neuroscience between 1999 and 2001. He and Rusty Gage were responsible for leading the Society through a profoundly tumultuous period and then laying the foundation for its ongoing success, including the hiring of Marty Saggase as Executive Director.

Dennis W. Choi, MD, Ph.D. (ret’d)

Professor and Chair

Department of Neurology, Stony Brook University

Donald Price: great human being, esteemed scientist, and friend

I recall an incident in the 1990’s. Don, Marcelle Morrison-Bogorad (deceased), and Tony Phelps were coordinating an NIH-NIA filming of current advances in Alzheimer’s Disease. I was invited to describe my teams work with hippocampal imaging and the early in vivo Alzheimer diagnosis. The scene was set, cameras rolling, Don asking me questions, and I nervously trying to keep my answers short, accurate, and informative.

In preparation for this event, I asked the late Henry Wisniewski to reach into our brain bank to excise a complete hippocampus. So, during the filming I went to the small jar of formalin I brought with me from New York and scooped out my prop. Gingerly balanced between my thumb and forefinger, and midway through my description, the specimen slipped out of my gloved hand and ended up on the floor.

For a few seconds I was mortified until Don put his arm around my shoulders, tilted his head back, and heartedly laughed. What a relief to pick up the specimen, join the laughter, and start over.

Thanks Don for being there and for the many scientific and educational opportunities we shared.

Mony J. de Leon, Ed.D.

Professor of Neuroscience and Radiology

Weil Cornell Medicine

Don Price and Sam Sisodia were the first Alzheimer’s experts that I encountered after joining Paul Greengard’s lab at Rockefeller in 1986. They played pivotal roles in initiation of our program in APP metabolism and cell biology, in part by introducing Paul and me to Axel Unterbeck and Ram Ramabhadran, then at Molecular Therapeutics, Inc., in West Haven CT. Axel and Jie Kang were with Benno Muller-Hill in the collaboration with Colin Masters and Konrad Beyreuther that produced the Kang et al. *Nature* paper in 1987 that reported the full length cloning of APP. Don’s enthusiasm for rigorous science, his upbeat mood, and his wide smile were reliable sources of moral support for the next 3½ decades. The one quote of sage advice that I will always hear in Don’s voice was not about science per se but about fundraising: *“Just keep your hand out”.* That was typical Don. Pragmatic and direct. He is already missed.

Sam Gandy, M.D., Ph.D.

Mount Sinai Professor of Alzheimer's Disease Research,

Professor of Neurology and Psychiatry,

Icahn School of Medicine at Mount Sinai
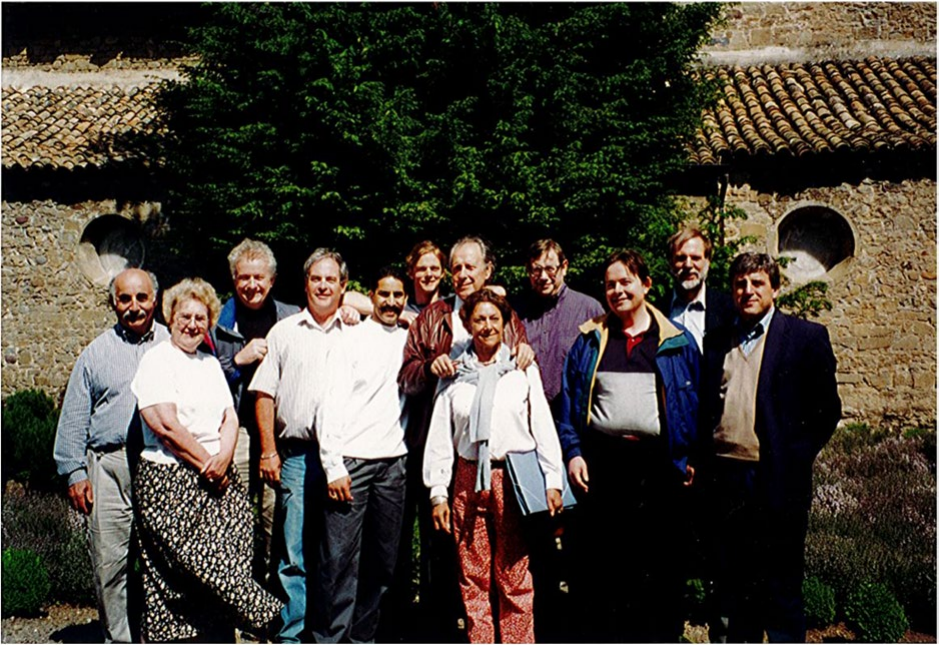


**1995** **:** **First Cajal Symposium Cajal’s birthplace: Petilla de Aragon**

Albert Aguayo, Don Price, Harry Orr, Jack Griffin, Greg Gasic, Kurt Fishbeck, Jose Lopez-Barneo

I am writing to extend my deepest condolences on the death of our long-time friend Dr. Donald L. Price. It is with heavy hearts that we mourn the loss of such a wonderful man and distinguished figure in the field of neuroscience. Throughout his remarkable career, Dr. Price made invaluable contributions to the study of neuropathology, leaving an indelible mark on the scientific community. His unwavering dedication, expertise, and ground breaking research have advanced our understanding of neurological diseases, especially Alzheimer’s Disease, paving the way for improved treatments and patient care.

Beyond his professional accomplishments, Don was well known for his compassion, good humour, mentorship, and unwavering commitment to his students and colleagues. His willingness to share his knowledge and guidance has inspired countless individuals and shaped the careers of many aspiring neuropathologists. During this difficult time, our thoughts are with his family, friends, and colleagues, as they go through this profound loss. May they find solace in knowing that his impact on the field of neurology will endure, and his memory will always be held dear by those whose lives he touched.

Albert Aguayo, MD, OC, FRSC

Emeritus Professor of Neuroscience

McGill University,

Montreal, Canada

In the early days when I first joined the Neuropathology Lab at Johns Hopkins, I always knew when Don Price was in town. He reveled in coming into the lab to see what new data or new finding had been revealed. Don brought boundless energy and great enthusiasm to science. He was fascinated by neurodegenerative disease of any sort and had limitless interests. Don’s enthusiasm was balanced by his unwavering dedication to rigor, particularly in studies involving neuropathologic techniques. Don had seen every type of artefact and knew every remedy to eliminate them. One of Don’s other great strengths was his sense of responsibility to support the career advancement of his more junior investigators. I am eternally grateful that the early days of my career were shaped by Don Price and my colleagues of the Neuropathology Laboratory he built.

David R. Borchelt, Ph.D.

CTRND

University of Florida
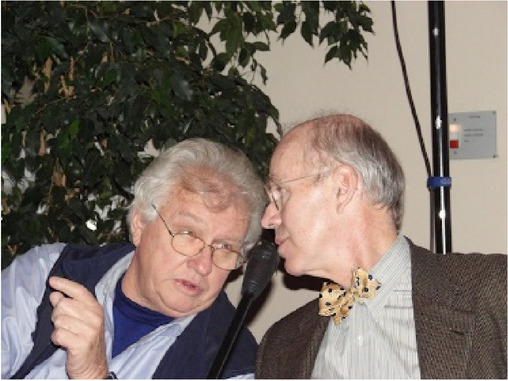


**2006**: **With John Growdon: Alzheimer’s 100**^**th**^** Anniversary (Tubingen).**

Don Price: Critic, Colleague and Friend

In 1982, I invited Don to assist in a mock site visit session, examining a Program Project grant proposal on Alzheimer disease before submission. Prior to his arrival, I had never met him as he had finished his training in Neurology and Neuropathology at the Massachusetts General Hospital several years before I began mine there. I figured we shared some common experiences with Ray Adams and E. P. Richardson and that he would be sensitive to our institutional plans. Further, he had begun to make important discoveries in Alzheimer disease and would be well positioned to advise us. Normally, mock site visitors toss some soft-pitch questions and conclude things are going well. With Don, it was the opposite: There was sharp criticism for much of our proposal – although there were a few good bits – and it was clear that we were behind the Alzheimer research curve and that we needed to up our game. This was my introduction to the most defining aspect of Don’s career: Insistence on the highest level of scientific research that he imposed on himself and on those who flocked to his laboratory.

In 1984, the National Institute on Aging put out an RFA for proposals to establish Alzheimer Disease Research Centers. With fresh money available there was considerable competition, and five Centers were approved, including the one at Johns Hopkins that he directed and the one in Massachusetts that I directed. As Directors we met often and shared information and plans regarding Alzheimer research. In working together, the Center structure fostered collegiality over criticism and competition. Over the ensuing years, during regular Center Director meetings and national and international meetings, the collegiality merged into genuine friendship as we discovered shared interests in literature, music and the importance of family. I was saddened by the disabilities that limited him in the last few years and know that his death deprives the Alzheimer community of one of its strongest voices. But I’m also aware that his legacy of scientific excellence carries on in the impressive cadre of trainees and in those who he did not formally train but who were influenced by him.

John H. Growdon, M.D

Professor of Neurology

Harvard Medical School

Director, Memory and Movement Disorders Unit,

Massachusetts General Hospital
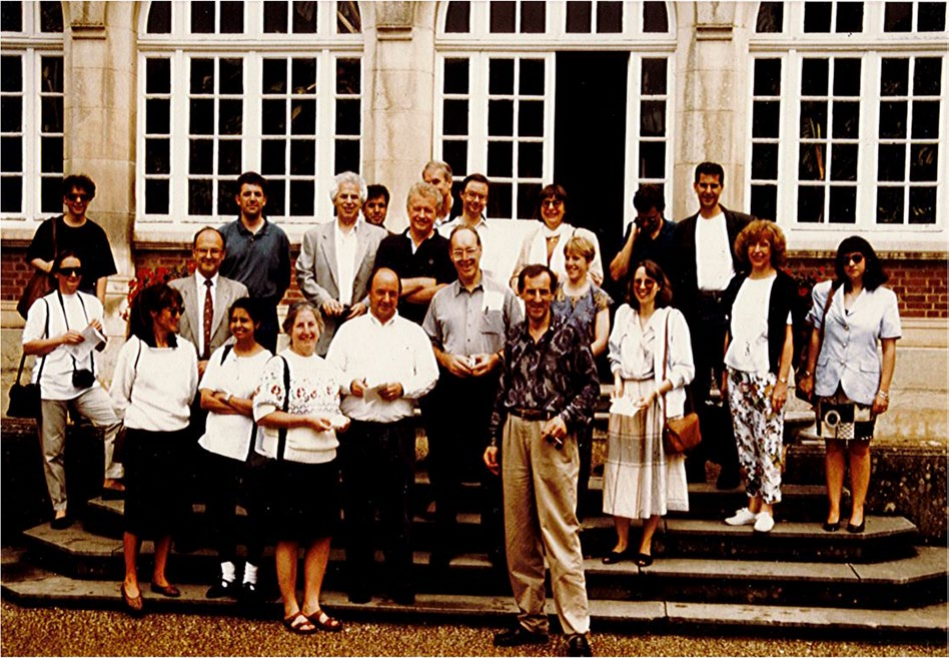


**1997 IPSEN mtg** **:** **Sam Gandy, Steve Younkin, Stanley Prusiner, Rudy Tanzi, Don Price, Colin Masters, Ashley Bush, Flint Beal, Konrad Beyreuther**

The late Donald L. Price was a good friend, a great and fair collaborator of us since 1990. He was exceptional in giving numerous credits to us for sending him and his coworkers our APP cDNA clone shortly after we isolated it in 1987. Don called one of us asking about its availability, and promised to keep us updated on its use and results, and he kept his word. The first results he and his coworkers obtained were the findings that APP undergoes fast axonal anterograde transport, which we published together in February 1990 (with Eddy Koo and Sam Sisodia). This was the first clear hint for a synaptic function of APP, a finding that was corroborated 30 years later. Four more papers followed together with Don as senior author in the same year. Unforgettable are Don’s reports on Rupert, the old rhesus monkey, his amyloid pathologic changes and his inability to eat bananas (papers published with Lee Martin, Larry Walker and Linda Cork). In the following years, we invited Don to numerous scientific meetings, including the Berlin-Dahlem conferences in Berlin, and he invited us to several in return. Unforgettable are his scientific presentations about amyloid plaques and vascular deposits in aged human primates that were clearly cognitively impaired without having tangles. That amyloid is the key player in Alzheimer`s disease was shown this year by the De Strooper lab, again many years (exactly 32) after Don’s seminal findings in primates. We enjoyed numerous dinners with Don and his wife Helen where we talk about Alzheimer’s disease and “God and the world”. Don was an exceptionally bright person, talking to him was always a pleasure, and stimulating. We miss a great colleague and friend. Above all, we thank Don for being our friend.

Konrad Beyreuther Dr. Dr. h.c., Seniorprofessor distinctus

Director Network Aging Research (NAR)

Heidelberg University

...and

Colin L Masters MD

Professor

Florey Institute and The University of Melbourne

I was never a member of the Don Price lab at JHU, but as a postdoctoral fellow at the Gerontology Research Center, NIA, in Baltimore, I visited often and worked closely with members of the lab. At that time, the lab included brilliant scientists such as Lary Walker, Sam Sisodia, Phil Wong, Linda Cork, Juan Troncoso, Vasillis Koliatos, Cheryl Kit, Lee Martin, and many others. Each time I visited, I sensed a vibrant environment on the front lines of Alzheimer's disease. It was the time when the first APP transgenic mice were developed, and although this did not happen in the Price lab, I felt that Don was the eminence in deciding whether the Alzheimer pathology in the mouse model was real or an artifact (Jucker et al., 1992). This was when α-secretase was identified and Alzheimer's pathology was described in old, nonhuman primates, all at around the same time in the Price lab. As a young postdoc, I always felt welcome in Don's lab. It was a wonderful time in my career and undoubtedly one of the best times in my scientific life. I also remember Don attending the 2006 AD centennial meeting that we organized in Tübingen. Although I didn’t get the chance to meet Don again in the last 15 years, I miss him.

Mathias Jucker, Ph.D.

Professor

Director at the Hertie Institute for Clinical Brain Research

The University of Tübingen

I so admired Don- the breadth and depth of his knowledge, the impact of his science, and his unswerving dedication to his students and trainees. Everything seemed to come effortlessly to him, and yet he displayed no ego. It was all about helping the other. As I rose through the ranks, I always welcomed our conversations in the halls. He seemed to immediately grasp the challenge I was having, and to have a solution that I hadn’t thought of. Through these many conversations, Don became an incredible mentor, almost a father figure, to me. He is missed and fondly remembered.

Ralph H. Hruban, M.D.

Director The Sol Goldman Pancreatic Cancer Research Center at Johns Hopkins Hospital

Baxley Professor and Director, Department of Pathology

The Johns Hopkins University School of Medicine
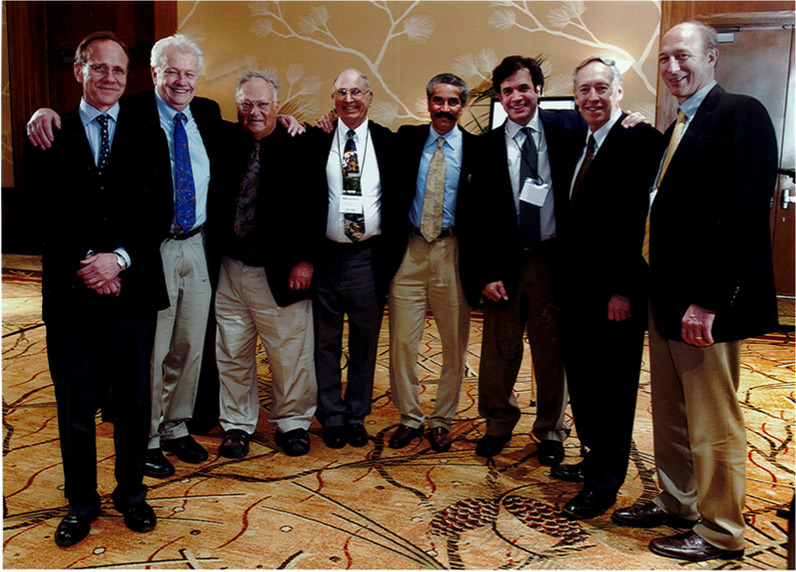


**25** ^**th**^ **Adler Symposium 2016: Rusty Gage, Don Price, Steve Heineman, John Adler, Sam Sisodia** **,** **Rudy Tanzi** **,** **Dennis Selkoe** **,** **David Holtzman**

During his illustrious career in biomedicine, I had the pleasure of interacting with Don Price over more than four decades. His deep knowledge of the pathology of the human brain, acquired beginning with his early training at MGH with some of the world’s premier neuropathologists, was a source of clarity for many students of brain disease, including me. Don had seen myriad cases of common and rare disorders, and he could be counted on to fill us in on details of how the brain changed with disease. He transmitted his knowledge succinctly and with warmth and humility. Beyond his understanding of how the brain changes with age and disease, Don was dedicated to imparting his knowledge to his many “trainees”, both those who had the privilege of formally working under him and the many of us who interacted with him at meetings and through animated conversations. Don taught neuropathology in many settings. I was a regular attendee at the Adler Foundation symposia each February at Scripps, and Don and his close colleagues organized these eclectic scientific sessions, where those lucky to be invited conversed freely about the thorny biological dilemmas we faced in deciphering Alzheimer’s disease and other complex disorders. Don had a charming, down-to-earth style – warm and avuncular – that made it easy to discuss and debate brain science with him. While he had well-grounded and firm opinions about how brain diseases like Alzheimer’s evolved, he was open to alternative points of view. He may not have expressed the level of enthusiasm for the ‘amyloid hypothesis’ that I did, but he could deliver his questions and concerns without a sense of dismissal. It was fun to debate him, always ending on a positive note …and that engaging smile of his.

Don was a dedicated mentor, and some of his most accomplished trainees are contributing to his collection. He had a lasting and highly salutary impact on his trainees. Don became a leader in neuroscience and helped organize innumerable small and large meetings. He was elected to the Presidency of the Society of Neuroscience, a particularly important and prestigious responsibility.

I had the opportunity to work on a few co-authored projects with Don and/or some of his gifted colleagues, including Eddie Koo, Linda Cork, Sam Sisodia, and others. Don’s interest in and knowledge of primate models was legendary, and I learned a lot about how closely (or sometimes not) lower primates followed human patterns of neuropathology. We all enjoyed his compellingly illustrated lectures on aged monkeys and he lessons he drew from them.

Don, we sorely miss your wisdom, humor and friendship as we continue to battle the diseases you cared about so deeply.

Dennis J. Selkoe, M.D.

The Vincent and Stella Coates Professor of Neurologic Diseases

Harvard Medical School

Co-Director, Center for Neurologic Diseases

Department of Neurology

Brigham and Women’s Hospital

Donald (Don) Price:

Don was a friend and a wonderful mentor. In 1973 while doing a fellowship in neuropathology in Boston, I came to down to Baltimore for career advice from Guy McKhann and Don. They both emphasized that for me to remain in academia, I needed to be a successful researcher. My medical training was in the UK, where research was not emphasized. To start my research training, I was offered a position as an instructor in neuropathology in Don’s division. Furthermore, I was handed a project studying the development of the optic nerve. To ensure my success with adequate time in the laboratory, my only commitment was half a day signing out cases. During those early days I met Don on a regular basis to discuss my research. He always had very exacting questions and suggestions that was interjected with some levity. Towards the end as an instructor his advice was that I should publish in excellent journals as what mattered was quality and not quantity of papers.

After completing the 2 years as an instructor and having my own funding, I switched back to combining clinical work with research in neurochemistry. Throughout the many years, Don has always been interested in the work I was doing and offering advice not only on my projects but also about my career. After I moved away from Hopkins, Don always made certain to meet me at the annual neuroscience meeting where he was keen to hear about my research and importantly to hear how my career was developing. My success as clinician/scientist in large part was depended on Don whose advise and encouragement resulted in achieving my goals.

Gihan I. Tinekoon, MBBS

Emeritus Professor of Neurology

The Children's Hospital of Philadelphia

Perelman School or Medicine

The University of Pennsylvania

For me, the importance of meeting and then being mentored by Don Price is immeasurable. I have no doubt that any successes I have had as an academic neurologist and pathologist were and are influenced by Don’s tutelage. As a medical student who aspired to train first as a neurologist and then neuropathologist, Don was the ultimate role model. Always available for questions, and consistently a cheerleader for my work, Don created an atmosphere where young investigators were encouraged to think creatively and embrace the joy of science. He was a gem, and will be remembered as such.

Jonathan D. Glass, MD

Professor, Neurology and Pathology

Emory University School of Medicine

Don was a mentor at a critical time in my life and career. Serendipity and synchronicity brought us together around a story that understanding pathology could help people with dementia. Of course, many others laid the groundwork and participated in a team effort to describe the Nucleus Basalis of Meynert’s role in “Alzheimer’s” as in other conditions. His personal guidance and skill in grantsmanship led me to many grant awards including Sloan and McKnight. He helped me develop our bank and expand it to include others in town, including the BLSA, and around the world. We moved into other diseases and other brain areas looking for those illusive brain/behavior correlations. And of course, he guided me into my move to Cleveland to start a center there.

But I remember most the search with Linda and others for those animal models. And Don’s obsession with swimming. And the occasional lapse into fun (😊) when, for example, for his birthday we presented him a book with photographic evidence that loss of cells in the NBM caused baldness.

I did not see Don so much after the move. I visited Hopkins for an event where we discussed the challenges of the pharmaceutical industry. And later some of us put on a conference with the History of Medicine Institute with Peter Rabins and others in the Psychiatry Department with whom I kept closer contact. I am not sure how he felt about my broadening interests and growing critiques of the dementia field. But without the strong foundation of my training with Don and others at Hopkins I would not have been so (in)effective at being a troublemaker!

Peter J. Whitehouse, M.D., Ph.D.

Professor of Neurology, Psychiatry, Cognitive Science, Neuroscience, and Organizational Behavior

Case Western Reserve University

As a neurology resident, I first met Don in the early 1970s, when he came to Hopkins and was considering a move here. We presented him the complicated clinical story of a patient with an acute stroke (no imaging then) and Don first hypothesized what must have caused the patient's clinical course, then what the pathology had to be, and then he cut the brain and indeed the pathology was just as predicted, "a top of the basilar syndrome" with an embolus that had fragmented and produced bilateral occipital lobe infarctions. BRILLIANT!

Don and I subsequently commuted together to Hopkins hospital with Gihan Tennekoon, Mahlon Delong, and Jack Griffin, all junior faculty members. Don pushed me to meet him at the Columbia swimming pool at 530:600 in the morning on a regular basis.

While I chose a different scientific focus in academic neurology, Don always encouraged me in my career path. And an important piece of advice he gave to me, "Family First", is something I have relayed to my mentees ever since. Living close to Don and Helen in his last days in Columbia, gave me the opportunity to visit and talk with him about the department, the faculty, movies and books. What a friend and mentor and outstanding neuroscientist.

David Zee, M.D.

Professor of Neurology, Otolaryngology-Head and Neck Surgery, Ophthalmology and Neuroscience

The Johns Hopkins University School of Medicine

I was tremendously fortunate to have someone like Don as a mentor. Every meeting/discussion with him left you energized to tackle ongoing challenges and explore new questions. He seamlessly transitioned between neuroscience, therapeutic strategies, literature, and Japanese cinema. I was able to meet him and Helen just before the covid lockdown. He was ever curious, as always, about what was going on in the field, incredibly kind and generous with his time, and happy to be close to his family in South Carolina. He will be deeply missed by all those whose lives have been meaningfully enriched thanks to his mentorship.

Rishab Shyam, Ph.D.

Founder, Something New